# Aggregation-Induced Emission: Lighting Up hERG Potassium Channel

**DOI:** 10.3389/fchem.2019.00054

**Published:** 2019-02-08

**Authors:** Xiaomeng Zhang, Tingting Liu, Qi Li, Minyong Li, Lupei Du

**Affiliations:** Key Laboratory of Chemical Biology (MOE), Department of Medicinal Chemistry, School of Pharmacy, Shandong University, Jinan, China

**Keywords:** AIE light-up probes, hERG channel, cell imaging, fluorophore, pharmacophore

## Abstract

Based on the scaffold of astemizole and E-4031, four AIE light-up probes (**L1–L4**) for Human Ether-a-go-go-Related Gene (hERG) potassium channel were developed herein using AIE fluorogen(TPE). These probes showing advantages such as low background interference, superior photostability, acceptable cell toxicity, and potent inhibitory activity, which could be used to image hERG channels at the nanomolar level. These AIE light-up probes hoped to provide guidelines for the design of more advanced AIE sensing and imaging hERG channels to a broad range of applications.

## Introduction

The potassium channels encoded by the hERG (human ether-a-go-go related gene) mediate the rapidly activating delayed rectifier K+ current (I_Kr_), which plays a key role in repolarization of the ventricular action potential (Perrin et al., 2008). A number of drugs were withdrawn from the market because of their blockade on the hERG channel, such as Cissapride, Tefenadine, Astemisole, and Grepafloxacin (Roden, 2004; Du et al., 2007; Yamakawa et al., 2012). They can induce long QT syndrome, which may degenerate into ventricular fibrillation and sudden death (Roden, 2004; Babcock and Li, 2013). Therefore the study of hERG inhibition has become an important part of modern safety pharmacology. Today, FDA guidelines expect that all drugs should be measured the affinity with hERG channel to evaluate their cardiotoxicity (Brown, 2004). Recently, many studies have shown that various cancer cell lines express hERG channels, whereas, corresponding normal cell lines do not express significant hERG protein, such as neuroblastoma, breast cancer, and colon cancer cells (Bianchi et al., 1998; Cherubini and Crociani, 2000; Pillozzi et al., 2002). hERG channels are in connection with some progresses, such as increasing tumor cell proliferation, invasion and lymphatic spread, reducing cell differentiation (Jehle et al., 2011). In these tumor cells, the hERG protein can be used as a biomarker for malignant transition.

In order to analyze the hERG channels, there is a stringent demand for a simple and safe method to image hERG channels. Such studies will conduce to a better understanding of the role of hERG channels in cancer cells and further improve the diagnosis and treatment of cancer. Among imaging technologies, fluorescence imaging is considered as one of the most significant methods in the medical research and life science field. Using fluorescence imaging, it is possible to directly visualize hERG channels. What counts is that fluorescent probe is a significant factor in fluorescence imaging. Nowadays, there have various fluorescent probes using in living cells and biological system by scientists from the fluorescence imaging field (Liu et al., 2016, 2017). However, many fluorescent probes show different fluorescent behaviors in dilute and concentrated solutions. The fluorescence intensity is often weakened or quenched at high concentrations, which is mechanistically related with the “formation of aggregates,” a phenomenon widely known as “aggregation-caused quenching” (ACQ) (Ding et al., 2013). Although scientists have made great efforts to overcome this phenomenon, ACQ effect is still an obstacle to the application of fluorescent probes to image biological target.

Tang's group found a novel fluorescent effect in 2001, a phenomenon named as “aggregation-induced emission,” (AIE) which was completely opposite to the ACQ effect. They discovered there was no fluorescence when hexaphenylsilole (HPS) was dissolved in a fine solvent but there was strong fluorescence when it was dissolved in a poor solvent (Hong et al., 2011). When hexaphenylsilole is in free state, intramolecular rotation occurs, and there is no emission, but intramolecular rotation is restricted when it is in aggregated state, leading to fluorescence. Since then, quite a number of AIEgens have emerged and have been used in biological imaging, chemical sensing, smart materials and optoelectronic devices. Among the AIEgens, tetraphenylethene (TPE) derivatives (Shi et al., 2012; Ding et al., 2013) have been widely applied in rational design of AIE probes. AIE light-up probes offer superior photostability and higher signal reliability comparing with conventional probes because of their higher resistance to photobleaching (Wang et al., 2016b).

## Results and Discussion

### Chemistry

**Scheme 1:**
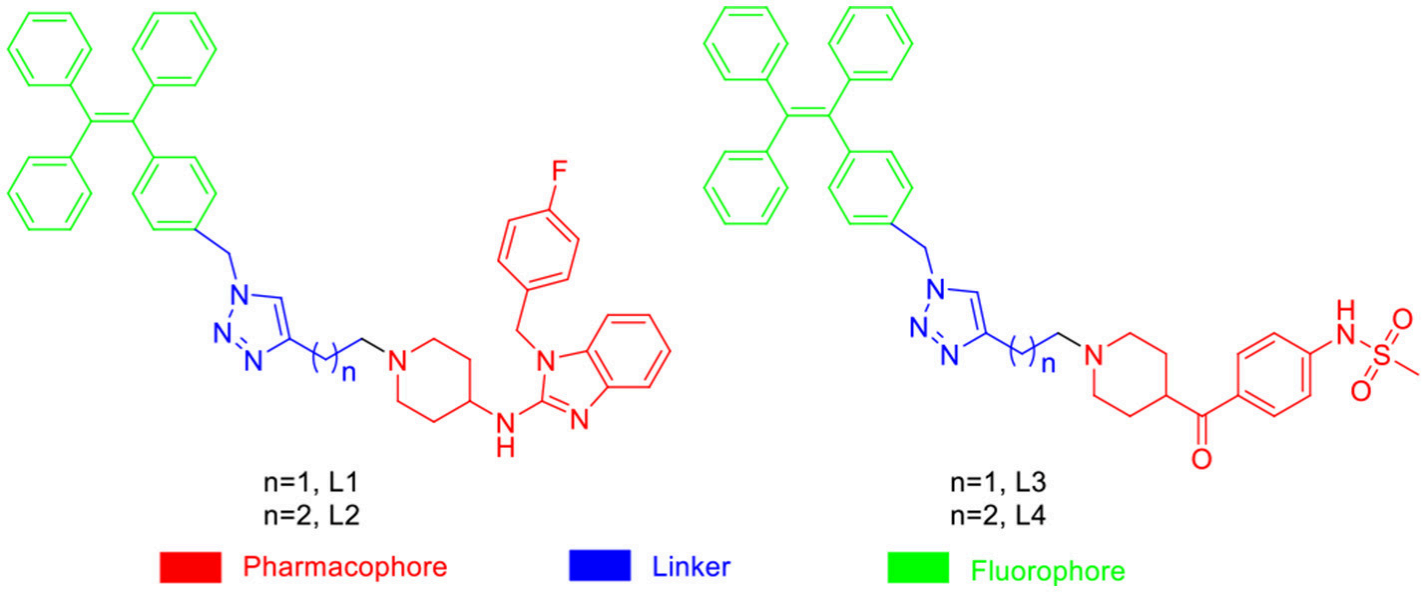
**Design Strategy of the Fluorescent probes.**.

**Scheme 2:**
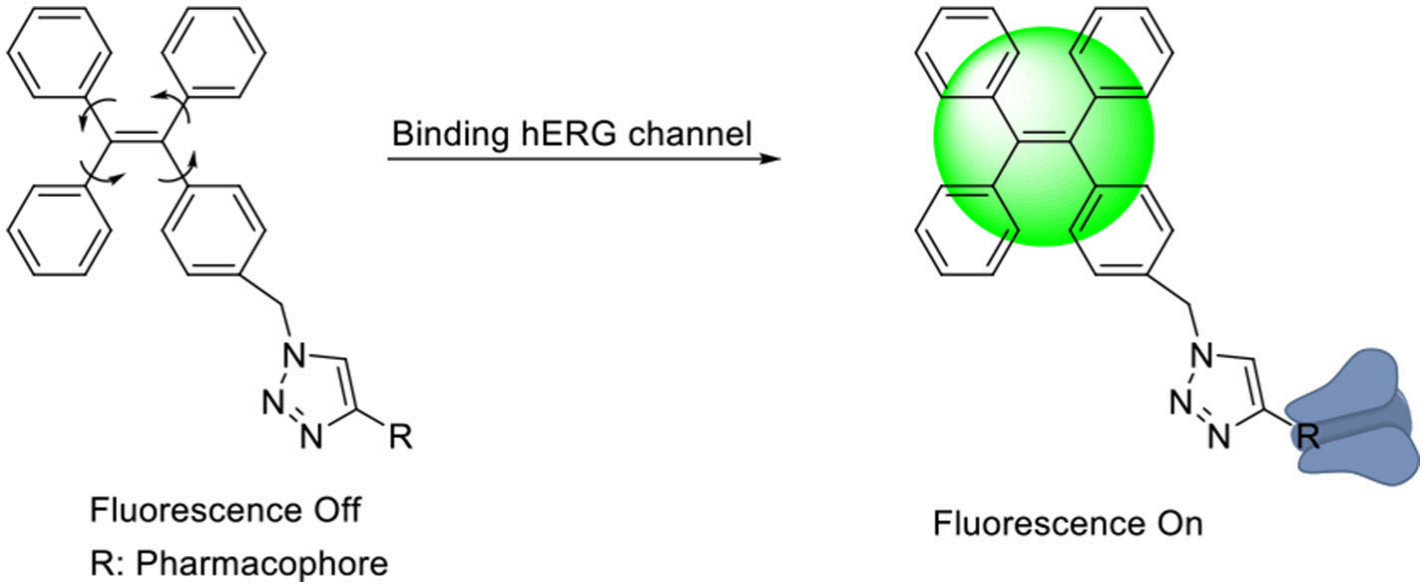
**The “Off-On” Mechanism of the Fluorescent Probes.**.

In general, the typical fluorescent probe consists of three parts: a fluorophore, a pharmacophore, and a linker (Cohen et al., 2002; Chen et al., 2014; Mizukami et al., 2014). In consideration of good fluorescent properties of TPE, it has been chosen as the fluorophore in the rational design for hERG channels probe. For pharmacophore, Astemizole and E-4031, the potent inhibitors of hERG channel, were chosen (Yamakawa et al., 2012; Wang et al., 2016a). For purpose of keeping the inhibitory activity of the probe, the major interaction sites of inhibitor's bonding were retained. Then the “click” reaction between the azoyl group on the fluorescence group and the acetylene group on the recognition group was carried out by copper (I) in order to generate the turn-on probe (Liang et al., 2015). The small molecule fluorescent probes (**L1–L4**) for the hERG channels were illustrated in Scheme 1. When the probe is in freestate, the benzene ring can rotate freely, and the molecules in excited state release energy in a nonradiative manner. However, the free rotation of the benzene rings is limited after the probe binding hERG channel, and the excited molecules mostly release energy as fluorescence (Scheme 2).

### Spectroscopic Properties of the Probes

**Figure 1:**
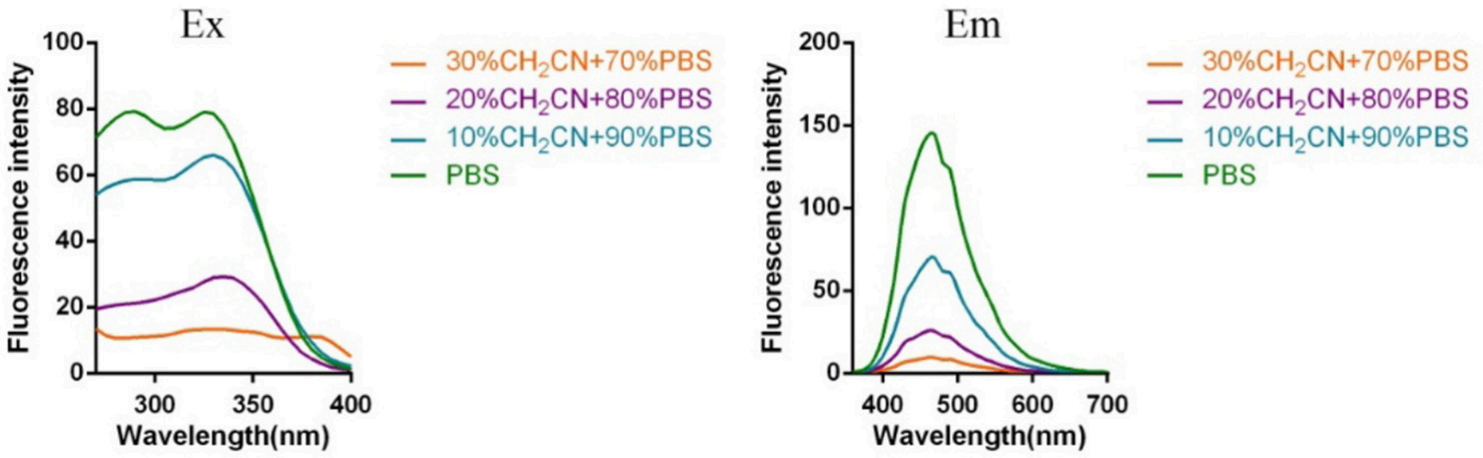
Fluorescent excitation (the emission wavelength was 465 nm) and emission spectra (the excitation wavelength was 325 nm) of probe **L1** in solution with different ratio of acetonitrile and PBS.

**Figure 2:**
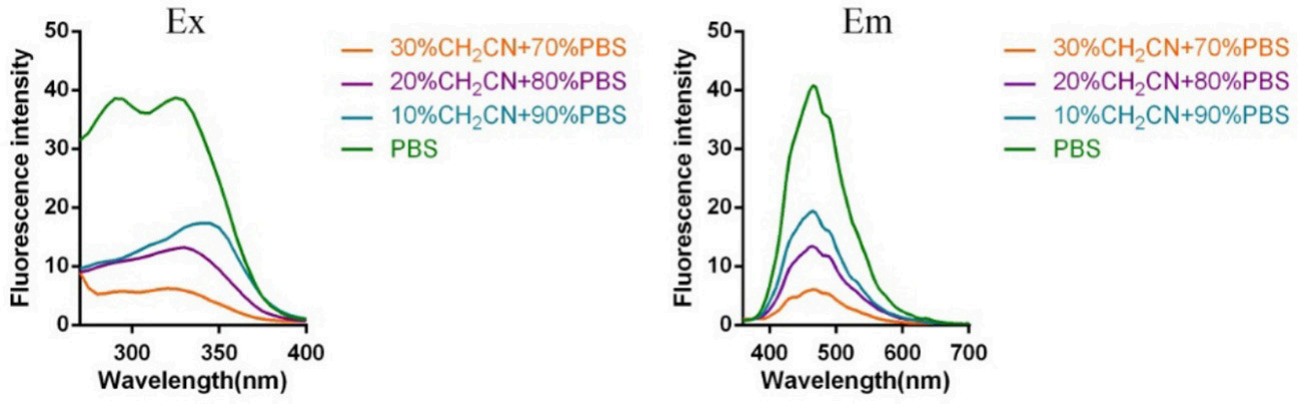
Fluorescent excitation (the emission wavelength was 465 nm) and emission spectra (the excitation wavelength was 325 nm) of probe **L2** in solution with different ratio of acetonitrile and PBS (Fluorescent excitation and emission spectra of probe **L3** and **L4** can be found in Supplementary Material).

Subsequently, the spectroscopic properties of the probes (**L1, L2, L3, L4**) were measured in 10 μM solution in PBS (pH = 7.4). The results indicated that all probes had similar fluorescent properties because of the same fluorophore (Table 1, Figures 1, 2).

**Table 1:** Photophysical properties of synthesized probes.

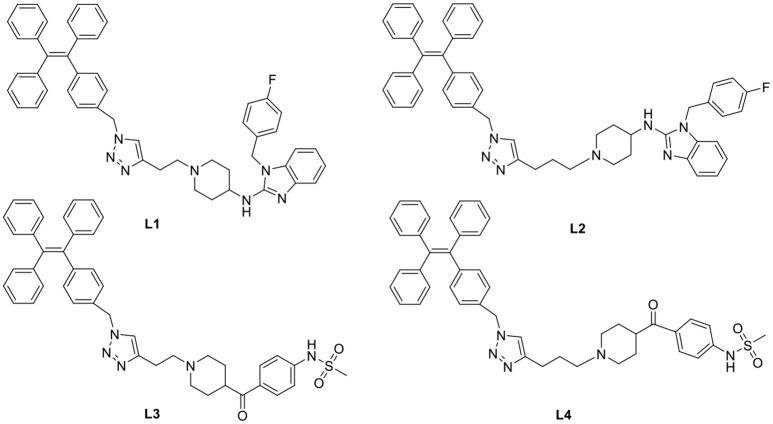				
**Compd**	**λ_max_(nm)**	**λ_ex_(nm)**	**λ_em_(nm)**	**Φ (%)**
**L1**	331	325	465	23
**L2**	329	325	465	25
**L3**	337	330	465	29
**L4**	336	330	465	25

### Binding Affinity of Probes

Afterwards, the inhibitory activities of the probes against the hERG potassium channel were evaluated utilizing radio-ligand binding assays by hERG transfected HEK293 cells. The results showed that probe **L3** displayed best inhibitory activity against the hERG channel, and the calculated IC_50_ and K_i_ values are 0.32 and 0.18 nM, respectively, which are slightly lower than astemizole (11.25 and 6.32 nM). Probe **L4** also showed lower inhibitory activity than astemizole, with IC_50_ values of 1.05 nM. Probes **L1** and **L2** showed potent inhibitory activity against hERG channel, with IC_50_ values of 120.50 and 155.90 nM, respectively, although lower than that of astemizole (See Table 2).

**Table 2:** Inhibitory activity of the synthesized probes against the hERG potassium channel^a^.

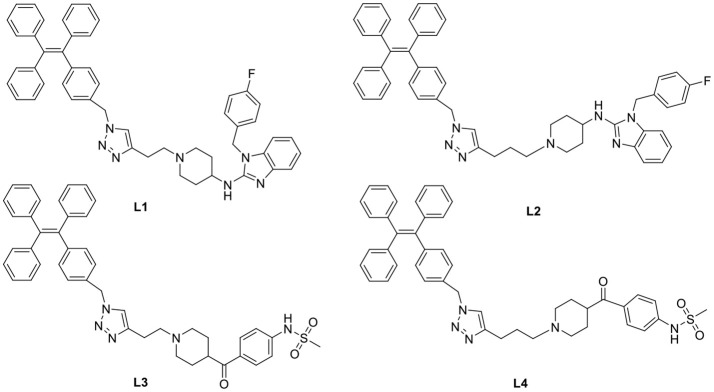		
**Compd**	**IC**_**50**_ **(nM)**	$K_{i}^{b}$ **(nM)**
**L1**	102.50	57.58
**L2**	155.90	87.58
**L3**	0.32	0.18
**L4**	1.05	0.59
Astemizole	11.25	6.32

*^a^see supporting information. ^b^the inhibition constant (K_i_) was calculated from each IC_50_ value using the Cheng–Prusoff equation*.

### Cytotoxicity Assay

The cytotoxicity of these probes was evaluated by CCK-8 assays using hERG transfected HEK293 cells. The results indicated that the IC_50_ of probes **L1–L4** were 3.55 ± 0.28, 2.43 ± 0.12, and 7.03 ± 0.14 μM in hERG-HEK293 cells (See Table 3).

**Table 3:** Cytotoxicity results for probes.

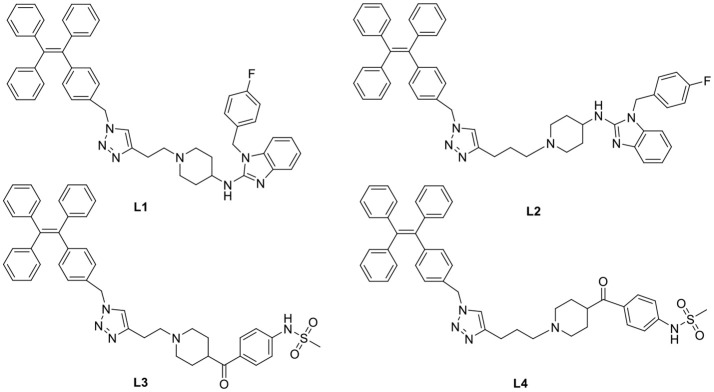	
**Compd**	**IC**_**50**_ **(μM)**
**L1**	20.74 ± 0.68
**L2**	8.56 ± 0.32
**L3**	39.99 ± 1.94
**L4**	17.37 ± 1.06
Astemizole	17.37 ± 1.07

### Fluorescent Image Assay

**Figure 3:**
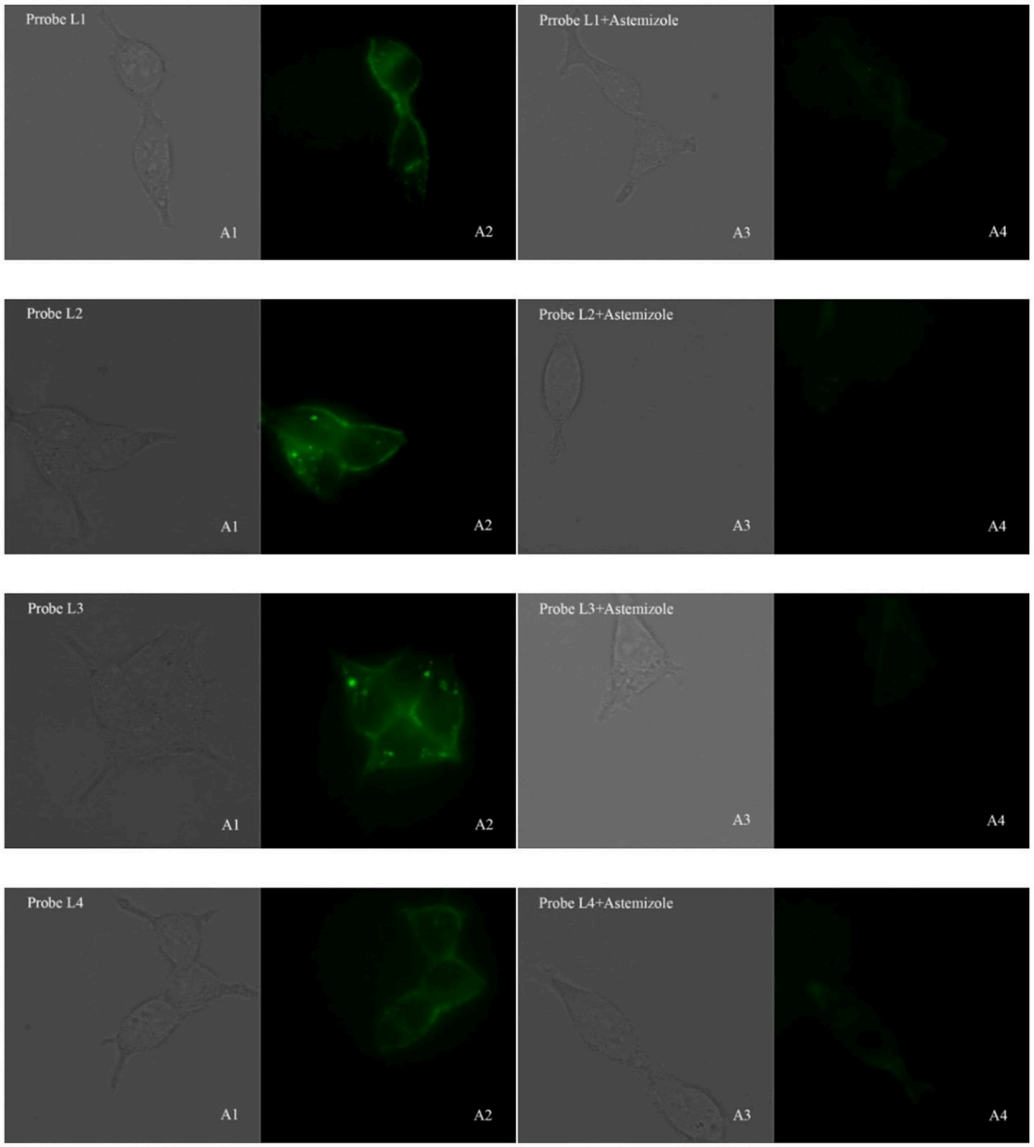
Fluorescence microscopy imaging of hERG transfected HEK293 cells incubated with 5 μM probe **L1**, 5 μM probe **L2**, 1 μM probe **L3**, 5 μM probe **L4** (A1, bright field; A2, GFP channel), respectively. The imaging of inhibition of the hERG channels was accomplished by incubating astemizole (50, 50, 10, 50 μM) with probe **L1** (5 μM), **L2** (5 μM), **L3** (1 μM), and **L4** (5 μM) (A3, bright field; A4, GFP channel). All cells were incubated with each probe at 37°C for 10 min and washed immediately. The background was adjusted by ImageJ software. Imaging was performed using a Zeiss Axio Observer A1 microscope with a 63 × objective lens. 
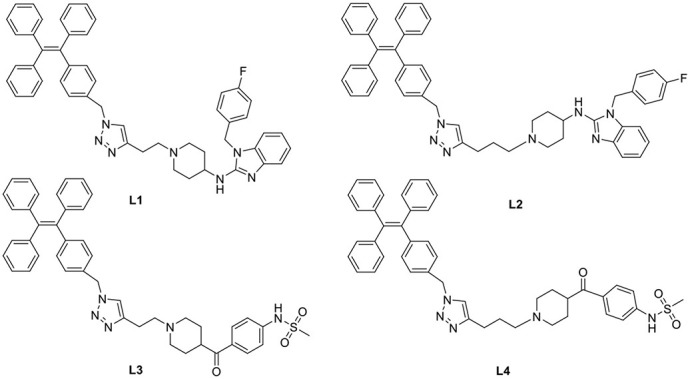
.

In consideration of their good fluorescent properties, acceptable cell toxicity, and potent inhibitory activity, probes **L1–L4** were utilized to image living cell in order to assess the capability of our probes for screening hERG channel. hERG transfected HEK293 cells were used to the imaging of probes **L1–L4** for hERG channels. Before we used probe to image cells, cell autofluorescence, and the effect of astemizole on cell autofluorescence were conducted (see Supplementary Material). The imaging results make clear that the autofluorescence of cells could be negligible in both the presence and absence of astemizole (Figure S9), which indicated the autofluorescence of the cells and astemizole would not interfere with the imaging of cells using probes **L1–L4**. The fluorescent imaging results demonstrated that these probes can label hERG-HEK293 cells with rapid responses and strong fluorescence to image hERG channels (Figure 3). Meanwhile, we chose a potent inhibitor of the hERG channels (astemizole) to incubate the cells with each probe. The fluorescence intensity was significantly decreased when the cells were co-incubated with astemizole and probe because of the inhibition of hERG channels by astemizole, which indicated that the hERG channels can be selectively labeled by the probe. Particularly, a complex washing procedure is not required because of their turn-on mechanism, which facilitated the experimental process. In conclusion, the results indicated that these probes all possess superior selectivity for hERG channels and could be used in the detection of hERG potassium channel.

## Conclusions

AIEgens are an emerging class of fluorophore with unique photophysical properties whose applications are attracting increasing attention. We designed four fluorescent probe for hERG channels based on AIE effect, with good fluorescent properties and acceptable cytotoxicity. The affinity for the hERG channel showed that the probes **L1–L4** have higher affinity for the hERG channel, especially the probe molecule **L3** (IC_50_ = 1.05 nM, K_i_ = 0.59 nM), which has a stronger affinity for the hERG potassium channel than the positive drug astemizole (IC_50_ = 11.25 nM, K_i_ = 6.32 nM). The probes **L1–L4** can be utilized to the localization and visualization of hERG channel, and they have been successfully utilized to label the hERG channels in hERG- HEK293 cells at the nanomolar level. These probes are anticipated to set up a screening system for hERG channels. But two aspects are deemed essential for these AIE light-up probes for hERG channels, these AIE light-up probes used blue green emissive TPE fluorogens, which have limited application in *in vivo* studies that require deep penetration and low autofluorescence. One choice is to design red or FR/NIR emissive AIEgens with desirable functionalities. The alternative option is to choose red or FR/NIR emissive fluorophore which can greatly widen the scope of fluorescent probe for hERG channels *in vivo* applications.

## Author Contributions

XZ, ML, and LD conceived and designed the experiments. XZ, TL, and QL performed the experiments. XZ, ML, and LD analyzed the data. XZ wrote the manuscript. LD retouched the document.

### Conflict of Interest Statement

The authors declare that the research was conducted in the absence of any commercial or financial relationships that could be construed as a potential conflict of interest.
