# Supplementary material for: Aggregation-Induced Emission: Lighting Up hERG Potassium Channel
Source: Front Chem. 2019 Feb 8;7:54. doi: 10.3389/fchem.2019.00054 (PMC6375833; doi:10.3389/fchem.2019.00054)
Supplement: Supplementary file 1 [file Data_Sheet_1.docx]

Supplementary Material

Aggregation-Induced Emission: Lighting up hERG Potassium Channel

Xiaomeng Zhang^1^, Tingting Liu ^1^, Qi Li^1^, Minyong Li^1^ and Lupei Du^1*^

*** Correspondence:** Lupei Du: [dulupei@sdu.edu.cn](mailto:dulupei@sdu.edu.cn)

Table of Contents

[1. Materials and instruments 2](#_Toc530848647)

[2. Synthesis 2](#_Toc530848648)

[2.1 Synthesis of intermediates(Ding D, 2013;Liu et al., 2016) 2](#_Toc530848649)

[2.2 Synthesis of probes 7](#_Toc530848650)

[3. Fluorescent excitation and emission spectra of free probe L1-L4 10](#_Toc530848651)

[4. The quantum yields measurement 11](#_Toc530848652)

[5. hERG potassium channel inhibition assay 12](#_Toc530848653)

[6. Cytotoxicity 13](#_Toc530848654)

[7. Cell culture and fluorescence microscopy imaging 13](#_Toc530848655)

[8. ^1^H-NMR, ^13^C-NMR, ESI-HRMS, HPLC. 14](#_Toc530848656)

[9. Reference 21](#_Toc530848657)

**1. Materials and instruments**

All solvents and reagents available from commercial sources were used as received unless otherwise noted. Water used for the fluorescence studies was doubly distilled and further purified with a Mill-Q filtration system. Melting points were determined on an electrothermal melting point apparatus and were uncorrected. ^1^H NMR and ^13^C NMR were recorded on a Bruker 400 MHz NMR spectrometer. Mass spectra were performed by the analytical and the mass spectrometry facilities at Shandong University and Shandong Provincial Academy of Sciences. Absorption spectra and fluorescence spectra were obtained with a Thermo Varioskan microplate reader. Fluorescence imaging was performed using Zeiss Axio Observer A1 fluorescence microscopy.

**2. Synthesis**

## 2.1 Synthesis of intermediates([Ding D, 2013](#_ENREF_1);[Liu et al., 2016](#_ENREF_2))

Scheme S1. Synthesis route of the intermediates

**2-chloro-1-(4-fluorobenzyl)-1H-benzo[d]imidazole (1a)**

A mixture of 2-chlorobenzimidazole (2.5 g, 16.35 mmol) and potassium carbonate (1.31 g, 23.3 mmol) in acetonitrile (15 mL) were refluxed for 30 min. The solution was cooled to room temperature and then 4-fluorobenzyl bromide (4.65 g, 24.05 mmol) was added. The mixture was refluxed for another 5 h. Water was added and the mixture was extracted with dichlormethane. The combined organic layers were dried over Na_2_SO_4_, filtered, and concentrated in vacuo. The crude product was recrystallized in petroleum ether to afford **1a** as white solid in 47% yield. M.p.: 66-69 °C. ^1^H-NMR (400 MHz, DMSO-*d6*): δ ppm: 7.65-7.62 (m, 2H), 7.32-7.24 (m, 4H), 7.22-7.16 (m, 2H), 5.53 (s, 2H); ESI-MS: ([M+H]^+^): 261.2.

**Ethyl-4-((1-(4-fluorobenzyl)-1H-benzo[d]imidazol-2-yl)amino)piperidine-1-carboxylate (2a)**

A mixture of compound **1a** (1.5 g, 5.7 mmol) and ethyl 4-aminopiperidine-1-carboxylate (1.485 g, 8.55 mmol) were subjected to Schlenk tube (170 °C, 8 h). After completion of the reaction, the solid was dissolved in dichloromethane and concentrated in vacuo. The crude product was purified by column chromatography to afford **2a** as white solid in 37% yield. M.p.: 178-180 °C. ^1^H-NMR (400 MHz, DMSO-*d6*): δ ppm: 7.23-7.18 (m, 1H), 7.17-7.13 (m, 2H), 7.08-7.06 (m, 1H), 6.96 (td, *J_1_* = 8.0 Hz, *J_2_* = 4.0 Hz, 1H), 6.86 (t, *J* = 8.0 Hz, 1H), 6.66 (d, *J* = 8.0 Hz, 1H), 5.26 (s, 1H), 4.07-4.02 (m, 2H), 3.99-3.94 (m, 2H), 2.96 (s, 2H), 2.52-2.50 (m, 2H), 2.01-1.97 (m, 2H), 1.47-1.37 (m, 2H), 1.24-1.15 (m,3H); ESI-MS: ([M+H]^+^)：397.4.

**1-(4-fluorobenzyl)-N-(piperidin-4-yl)-1H-benzo[d]imidazol-2-amine hydrobromide (3a)**

A mixture of compound **2a** (1 g, 2.52 mmol) and 40% HBr aqueous solution (25 mL) were stirred at 100 °C for 16 h. Thereafter, solvent was evaporated in vacuo and the crude product was recrystallized in ethyl acetate to afford **3a** as white solid in 93% yield. M.p.: 261-263 °C. ^1^H-NMR (400 MHz, DMSO-*d6*): δ ppm: 9.05 (s, 1H), 8.06 (s, 2H), 7.52-7.50 (m, 1H), 7.43-7.41 (m, 2H), 7.39-7.35 (m, 2H), 7.33-7.26 (m, 2H), 7.24-7.18 (m, 2H), 5.51 (s, 2H), 4.06 (q, *J* = 8.0 Hz, 1H), 3.46 (d, *J* = 8.0 Hz, 2H), 3.03-2.97 (m, 2H), 2.20-2.15 (m, 2H), 1.99-1.84 (m, 2H); ESI-MS: ([M+H]^+^) : 325.4.

**1-methyl-4- (but-3-yn-1-ylsulfonyl)benzene (4a)**

In a 100 mL round bottom flask, 3-butyn-1-ol (1.00 g, 14.27 mmol) was dissolved in dichlormethane (15 mL), and triethylamine (2.89 g, 28.53 mmol) was added. 4-methylbenzenesulfonyl chloride (4.08 g, 21.40 mmol) was dissolved in dichlormethane (5 mL) and it was dropwise added to the reaction solution in ice bath condition. After the ice bath reaction for 2 h, move to the room temperature for 48 h. Water was added and the mixture was extracted with dichlormethane. The combined organic layers were dried over Na_2_SO_4_, filtered, and concentrated in vacuo. Thereafter, solvent was evaporated in vacuo and the crude product was recrystallized in petroleum ether to afford **4a** as colorless oil in 53% yield. ^1^H-NMR (400 MHz, DMSO-*d_6_*): δ ppm: 7.82-7.79 (m, 2H), 7.37 (d, *J* = 8.0 Hz, 2H), 4.13-4.09 (m, 2H), 2.58-2.54 (m, 2H), 2.46 (s, 3H), 1.98-1.97 (m, 1H); ESI-MS: ([M+Na]^+^): 247.3.

**1-methyl-4-(pent-4-yn-1-ylsulfonyl)benzene (5a)**

In a 100 mL round bottom flask, 4- pentyn-1-ol (0.80 g, 9.51 mmol) was dissolved in dichlormethane (15 mL), and triethylamine (1.92 g, 19.02 mmol) was added. 4-methylbenzenesulfonyl chloride (2.72 g, 14.27 mmol) was dissolved in dichlormethane (5 mL) and it was dropwise added to the solution in ice bath condition. After the ice bath reaction for 2 h, move to the room temperature for 48 h. Water was added and the mixture was extracted with dichlormethane. The combined organic layers were dried over Na_2_SO_4_, filtered, and concentrated in vacuo. Thereafter, solvent was evaporated in vacuo and the crude product was recrystallized in petroleum ether to afford **5a** as colorless oil in 43% yield. ^1^H-NMR (400 MHz, DMSO-*d_6_*): δ ppm: 7.81-7.78 (m, 2H), 7.50-7.48 (m, 2H), 4.10-4.06 (m, 2H), 2.77-2.76 (m, 2H), 2.43 (s, 3H), 2.20 (td, *J_1_* = 8.0 Hz, *J_2_* = 4.0 Hz, 2H), 1.78-1.71 (m, 2H); ESI-MS: ([M+H]^+^): 261.2.

**N-(1-(but-3-yn-1-yl)piperidin-4-yl)-1-(4-fluorobenzyl)-1H-benzo[d]imidazol-2-amine(6a)**

In a 100 mL round bottom flask, compound **4a** (221.00 mg, 986.70 μmol), compound **3a** (0.40 g, 986.70 μmol) and K_2_CO_3_ (341.00 mg, 2.47 mmol) were dissolved in acetonitrile (10 mL) and N, N-dimethylacetamide (4 mL). The mixture was heated to reflux for 12 h in 80 °C. After cooled down to room temperature, the reaction mixture was evaporated in vacuo and was extracted with DCM. The organic layer was collected and concentrated. The crude product was purified by silica-gel chromatography to give **6a** as white solid in 89% yield. M.p.: 125-127 °C. ^1^H-NMR (400 MHz, DMSO-*d_6_*): δ ppm: 7.23-7.17 (m, 3H), 7.15-7.14 (m, 2H), 7.06 (d, *J* = 8.0 Hz, 1H), 6.94-6.90 (m, 1H), 6.84-6.81 (m, 1H), 6.61 (d, *J* = 12.0 Hz,1 H), 5.26 (s, 2H), 3.74-3.72 (m, 1H), 2.48-2.46 (m, 2H), 2.34-2.30 (m, 2H), 2.10-2.05 (m, 2H), 1.96 (d, *J* = 12.0 Hz, 2H), 1.57-1.48 (m, 2H), 1.24 (s, 1H); ESI-MS: ([M+H]^+^) : 377.4.

**1-(4-fluorobenzyl)-N-(1-(pent-4-yn-1-yl)piperidin-4-yl)-1H-benzo[d]imidazol-2-amine(7a)**

In a 100 mL round bottom flask, compound **5a** (235.18 mg, 986.89 μmol), compound **3a** (0.40 g, 986.89 μmol) and K_2_CO_3_ (170.49 mg, 1.23 mmol) were dissolved in acetonitrile (15 mL) and N, N-dimethylacetamide (6 mL). The mixture was heated to reflux for 12 h in 80 °C. After cooled down to room temperature, the reaction mixture was evaporated in vacuo and was extracted with DCM. The organic layer was collected and concentrated. The crude product was purified by silica-gel chromatography to give **7a** as white solid in 22% yield. M.p.: 122-125 °C. ^1^H-NMR (400 MHz, DMSO-*d_6_*): δ ppm: 7.23-7.17 (m, 3H), 7.15-7.13 (m, 2H), 7.06 (d, *J*=8.0 Hz, 1H), 6.94 (t, *J* = 8.0 Hz, 1H), 6.84 (t, *J* = 8.0 Hz ,1H), 6.58 (d, *J* = 8.0 Hz,1 H), 5.26 (s, 2H), 3.75-3.73 (m, 1H), 2.84 (d, *J* = 12.0 Hz, 2H), 2.76-2.74 (m, 2H), 2.36-2.33 (m, 2H), 2.20-2.16 (m, 2H), 2.03-1.94 (m, 4H), 1.64-1.69 (m, 4H); ESI-MS: ([M+H]^+^): 391.4.

**N-(4-(1-acetylpiperidine-4-carbonyl)phenyl)methanesulfonamide(1b)**

In a 100 mL round bottom flask, 1-acetylpiperidine-4-carboxylic acid (2.00 g，11.68 mmol) was dissolved in 1, 2-dichloroethane (30 mL) in 40 °C. SOCl_2_(1.39 g，11.68 mmol) was dissolved in 1, 2-dichloroethane (5 mL) and it was dropwise added to the solution. After the addition, the temperature is raised to 65 °C and the reaction was stirred for 1 h. After cooled down to room temperature, N-phenylmethanesulfonamide (2.00 g, 11.68 mmol) was added in ice bath condition. Afterwards, AlCl_3_ (3.12 g, 3.36 mmol) was added into the reaction solution in ice bath condition. After the reaction solution stirred in ice bath condition for 30 min, the reaction solution was heated to reflux for 12 h in 80 °C. Crushed ice was then added, and the solution was extracted three times with DCM. The combined organic layers were dried over Na_2_SO_4_, filtered, and concentrated in vacuo. Thereafter, solvent was evaporated in vacuo and the crude product was recrystallized in petroleum ether to afford **1b** as light pink solid in 29% yield. M.p.: 211-214 °C. ^1^H-NMR (400 MHz, DMSO-*d_6_*): δ ppm: 10.34 (s, 1H), 8.01-7.97 (m, 2H), 7.32-7.28 (m, 2H), 4.40-4.38 (m, 2H), 3.67-3.59 (m, 1H), 3.22-3.15 (m, 1H), 3.12 (s, 3H), 2.75-2.68 (m, 1H), 2.00 (s, 3H), 1.80-1.74 (m, 2H), 1.56-1.46 (m, 1H), 1.38-1.28 (m, 1H); ESI-MS: ([M+H]^+^): 325.4.

**N-(4-(piperidine-4-carbonyl)phenyl)methanesulfonamide hydrochloride(2b)**

A mixture of compound **1b** (1.00 g, 3.08 mmol) and 30 mL hydrochloric acid solution (1 M) was stirred at 100 °C for 12 h. After cooled down to room temperature, the reaction solution was filtered to afford **2b** as light pink solid in 95% yield. M.p.: ＞270 °C. ^1^H-NMR (400 MHz, DMSO-*d_6_*): δ ppm: 10.42 (s, 1H), 9.03 (s, 1H), 8.43 (s, 1H), 8.01-7.98 (m, 2H), 7.33-7.30 (m, 2H), 3.74-3.66 (m, 1H), 3.32-3.28 (m, 2H), 3.12 (s, 3H), 3.07-2.98 (m, 2H), 1.94-1.89 (m, 2H), 1.81-1.70 (m, 2H); ESI-MS: ([M+H]^+^): 283.3.

**N-(4-(1-(but-3-yn-1-yl)piperidine-4-carbonyl)phenyl)methanesulfonamide(3b)**

In a 100 mL round bottom flask, compound **4a** (418.47 mg, 1.88 mmol), compound **2b** (0.60 g, 1.88 mmol) and K_2_CO_3_ (650.24 mg, 4.70 mmol) were dissolved in acetonitrile (15 mL) and N, N-dimethylacetamide (6 mL). The mixture was heated to reflux for 12 h in 80 °C. After cooled down to room temperature, the reaction mixture was evaporated in vacuo and was extracted with DCM. The organic layer was collected and concentrated. The crude product was purified by silica-gel chromatography to give **3b** as white solid in 21% yield. M.p.: 180-183 °C. ^1^H-NMR (400 MHz, DMSO-*d_6_*): δ ppm: 10.42 (s, 1H), 8.01-7.96 (m, 2H), 7.33-7.30 (m, 2H), 3.67-3.60 (m, 5H), 3.27-3.22 (m, 2H), 3.12 (s, 3H), 3.11 (t, *J* = 4.0 Hz, 1H), 2.79-2.70 (m, 2H), 2.00-1.84 (m, 4H); ESI-MS: ([M+H]^+^): 335.5.

**N-(4-(1-(pent-4-yn-1-yl)piperidine-4-carbonyl)phenyl)methanesulfonamide(4b)**

In a 100 mL round bottom flask, compound **5a** (373.73 mg, 1.57 mmol), compound **2b** (0.50 g, 1.57 mmol) and K_2_CO_3_ (541.87 mg, 3.92 mmol) was dissolved in acetonitrile (15 mL) and N, N-dimethylacetamide (6 mL). The mixture was heated to reflux for 12 h in 80 °C. After cooled down to room temperature, the reaction mixture was evaporated in vacuo and was extracted with DCM. The organic layer was collected and concentrated. The crude product was purified by silica-gel chromatography to give **4b** as white solid in 20% yield. M.p.: 191-193 °C. ^1^H-NMR (400 MHz, DMSO-*d_6_*): δ ppm: 10.41 (s, 1H), 10.18 (s, 1H), 8.00-7.96 (m, 2H), 7.34-7.30 (m, 2H), 3.69-3.61 (m, 1H), 3.56 (t, *J* = 12.0 Hz, 1H), 3.12 (s, 3H), 3.11-3.01 (m, 4H), 2.94-2.92 (m, 1H), 2.31-2.25 (m, 2H), 1.99-1.85 (m, 6H); ESI-MS: ([M+H]^+^): 349.4.

**1,2,2-triphenyl-1-(p-tolyl)ethan-1-ol(c1)**

In a 250 mL two necked round bottom flask, diphenylmethane (1.68 g, 10.00 mmol) was dissolved in 100 mL of distilled THF under N_2_. After the mixture was cooled to 0 °C, 5 mL of n-butyllithium was slowly added by a syringe. The mixture was stirred at 0 °C for 1 h. 4-methylbenzophenone (1.63 g, 8.30 mmol) was then added into the reaction mixture. The mixture was warmed to room temperature and stirred overnight. The reaction mixture was quenched with saturated NH_4_Cl solution and then extracted with DCM to afford **c1** as white solid in 86% yield. M.p.: 138-141 °C. ^1^H-NMR (400 MHz, DMSO-*d_6_*): δ ppm: 7.47 (t, *J* = 8.0 Hz, 5H), 7.40 (t, *J* = 8.0 Hz, 3H), 7.13-6.92 (m, 11H), 5.87 (s, 1H), 5.20 (s, 1H), 2.15 (s, 3H).

ESI-MS: ([M+Na]^+^): 387.4.

**(2-(p-tolyl)ethene-1,1,2-triyl)tribenzene(c2)**

Compound **c2** (0.50 g, 1.43 mmol) and p-toluenesulfonic acid (10.00 mg, 47.19 μmol) were dissolved into 10 mL of toluene. The mixture was heated to reflux for 12 h in 120 °C. After cooled down to room temperature, the reaction mixture was extracted with DCM. The organic layer was collected and concentrated. The crude product was purified by silica-gel chromatography using hexane as eluent to give **c2** as white solid in 74% yield. M.p.: 175-178 °C. ^1^H-NMR (400 MHz, DMSO-*d_6_*): δ ppm: 7.15-7.09 (m, 9H), 6.98-6.93 (m, 8H), 6.85 (d, *J* = 8.0 Hz, 2H), 2.20 (s, 3H); ESI-MS: ([M+NH_4_]^+^): 364.4.

**(2-(4-(bromomethyl)phenyl)ethene-1,1,2-triyl)tribenzene(c3)**

In a 50 mL round bottom flask, a solution of **c2** (0.70 g, 2.00 mmol), N-bromosuccinimide (0.40 g, 2.20 mmol), benzoyl peroxide (0.02 g, 0.08 mmol) in 12 mL of CCl_4_ was refluxed for 28 h. After the reaction was completed, the mixture was extracted with dichloromethane and water. The organic layers were combined, dried over magnesium sulfate, and removed under reduced pressure. The crude product was purified by silica-gel chromatography using hexane as eluent to give c3 as white solid in 49% yield. M.p.: 120-123 °C. ^1^H-NMR (400 MHz, DMSO-*d_6_*): δ ppm: 7.13-7.09 (m, 11H), 7.03-6.98 (m, 8H), 4.42 (s, 2H); ESI-MS: ([M+NH_4_]^+^): 442.4.

**(2-(4-(azidomethyl)phenyl)ethene-1,1,2-triyl)tribenzene(c)**

In a 50 mL two-neck round-bottom flask, **c3** (0.20 g, 0.50 mmol) and sodium azide (48.00 mg, 0.75 mmol) were dissolved in DMSO (12 mL) under N2. The mixture was stirred at room temperature overnight. A large amount (100 mL) of water was then added, and the solution was extracted three times with DCM. The organic layers were combined, dried over magnesium sulfate, and concentrated. The crude product was purified by silica gel chromatography to give **c** as white solid in 75% yield. M.p.: 105-108 °C. ^1^H-NMR (400 MHz, DMSO-*d_6_*): δ ppm: 7.04-7.02 (m, 9H), 6.97-6.94 (m, 10H), 4.18 (s, 2H); ESI-MS: ([M+ NH_4_]^+^): 405.5.

**2.2 Synthesis of probes**

2.2.1 Synthesis of probe **L1**([Ding D, 2013](#_ENREF_1))

In a 50 mL two-neck round-bottom flask, compound **6a** (60.00 mg, 137.81 μmol) and compound c (53.4 mg, 137.81 μmol) were dissolved in a mixture of t-BuOH/H_2_O solution (v/v = 2/1; 36 mL), and the reaction was shaken for a few minutes to obtain a clear solution. The “click” reaction was initiated by sequential addition of the solution of sodium ascorbate (0.1 M, 5.4 mL) and CuSO_4_ (0.1 M, 1.5 mL) in water. The reaction was continued with stir at 50 °C for another 1 h. The reaction mixture was evaporated in vacuo and was extracted with DCM. The organic layer was collected and concentrated. The crude product was purified by silica-gel chromatography, then added ethyl acetate saturated with hydrogen chloride to give probe **L1** as yellow-brown solid in 29% yield. M.p.: 241-243 °C. ^1^H-NMR (400 MHz, DMSO-*d_6_*): δ ppm: 14.27 (s, 1H), 11.26 (s, 1H), 9.50 (s, 1H), 8.02 (s, 1H), 7.51 (d, *J* = 12 Hz, 1H), 7.42-7.37 (m, 3H), 7.31-7.18 (m, 4H), 7.15-7.09 (m, 9H), 7.06 (d, 2H, *J* = 12 Hz), 6.99-6.94 (m, 8H), 5.57 (s, 2H), 5.51 (s, 2H), 4.14 (s, 1H), 3.71-3.68 (m, 2H), 3.43-3.31 (m, 2H), 3.23-3.19 (m, 2H), 3.07-3.04 (m, 2H), 2.24-2.22(m, 4H).

^13^C-NMR (100 MHz, DMSO-*d_6_*): δ ppm: 149.38, 143.45, 141.38, 140.42, 134.60, 131.39, 131.04, 131.01, 129.88, 129.79, 129.71, 128.37, 128.32, 128.26, 127.71, 127.15, 127.04, 124.20, 123.78, 123.46, 116.23, 116.02, 112.12, 111.03, 55.01, 52.86, 51.35, 49.94, 45.30, 28.87, 20.66.

ESI-HRMS: ([M+H]^+^) calcd for C_50_H_46_FN_7_: 764.3871, found: 764.3821.

HPLC, tR = 6.312 min, mobile phase: methanol-water (90:10, v/v), λ = 270 nm.

2.2.2 Synthesis of probe **L2**([Ding D, 2013](#_ENREF_1))

In a 50 mL two-neck round-bottom flask, Compound **7a** (151.15 mg，390.00 μmol) and compound c (150.00 mg，390.00 μmol) were dissolved in a mixture of t-BuOH/H_2_O solution (v/v = 2/1; 36 mL), and the reaction was shaken for a few minutes to obtain a clear solution. The “click” reaction was initiated by sequential addition of the solution of sodium ascorbate (0.1 M, 15.0 mL) and CuSO_4_ (0.1 M, 4.3 mL) in water. The reaction was continued with stir at 50 °C for another 1 h. The reaction mixture was evaporated in vacuo and was extracted with DCM. The organic layer was collected and concentrated. The crude product was purified by silica-gel chromatography, then added ethyl acetate saturated with hydrogen chloride to give probe **L2** as yellow-brown solid in 5% yield. M.p.: 155-157 °C.

^1^H-NMR(400 MHz, DMSO-*d_6_*): δ ppm: 14.40 (s,1H), 11.09 (s,1H), 9.50 (s,1H), 7.97 (s, 1H), 7.51 (d, 1H, *J* = 12 Hz), 7.41-7.37 (m, 3H), 7.30-7.17(m, 4H), 7.15-7.09 (m, 9H), 7.06-7.04 (m, 2H), 6.99-6.94 (m, 8H), 5.57 (s, 2H), 5.48 (s, 2H), 4.16 (s, 1H), 3.62-3.59 (m, 2H), 3. 15-3.10 (m, 2H), 3.03-3.00 (m, 2H), 2.71-2.67( m, 2H), 2.23-2.14 (m, 4H), 2.10-2.08 (m, 2H).

^13^C-NMR (100 MHz, DMSO-*d_6_*): δ ppm: 149.36, 146.20, 143.50, 143.46, 143.37, 141.36, 140.43, 134.69, 131.39, 131.04, 131.01, 129.86, 129.78, 129.72, 128.37, 128.32, 128.26, 127.68, 127.14, 127.03, 124.17, 122.97, 116.22, 116.01, 112.11, 111.01, 55.79, 52.81, 51.29, 49.98, 45.30, 28.83, 23.61, 22.81.

ESI-HRMS: ([M+H]^+^) calcd for C_51_H_48_FN_7_: 778.4028, found: 778.4027.

HPLC, tR = 6.030 min, mobile phase: methanol-water (91:9, v/v), λ = 270 nm.

2.2.3 Synthesis of probe **L3**([Ding D, 2013](#_ENREF_1))

In a 50 mL two-neck round-bottom flask, Compound **3b** (130.00 mg，388.72 μmol) and compound c (165.68 mg，427.59 μmol) were dissolved in a mixture of t-BuOH/H_2_O solution (v/v = 2/1; 36 mL), and the reaction was shaken for a few minutes to obtain a clear solution. The “click” reaction was initiated by sequential addition of the solution of sodium ascorbate (0.1 M, 15.0 mL) and CuSO_4_ (0.1 M, 4.3 mL) in water. The reaction was continued with stir at 50 °C for another 1 h. The reaction mixture was evaporated in vacuo and was extracted with DCM. The organic layer was collected and concentrated. The crude product was purified by silica-gel chromatography, then added ethyl acetate saturated with hydrogen chloride to give probe **L3** as yellow-brown solid in 16% yield. M.p.: 56-58 °C.

^1^H-NMR(400 MHz, DMSO-*d_6_*): δ ppm: 10.67 (s,1H), 10.44 (s, 1H), 8.03-7.97 (m, 3H), 7.34 (d, 2H, *J* = 8.0 Hz), 7.15-7.09 (m, 9H), 7.06-7.04 (m, 2H), 6.98-6.94 (m, 8H), 5.50(s, 2H), 3.67-3.60 (m, 3H), 3.37-3.34 (m, 2H), 3.19-3.10 (m, 7H), 1.97-1.94 (m, 4H).

^13^C-NMR (100 MHz, DMSO) δ 199.85, 143.80, 143.45, 143.42, 143.01, 141.38, 140.41, 134.58, 131.39, 131.04, 131.04, 131.01, 130.62, 128.32, 128.27, 127.71, 127.04, 123.47, 118.08, 60.22, 55.31, 52.84, 51.54, 26.20, 21.24, 20.49, 14.56.

ESI-HRMS: ([M+H]^+^) calcd for C_44_H_43_N_5_O_3_S: 722.3159, found: 722.3167.

HPLC, tR = 6.120 min, mobile phase: methanol-water (93:7, v/v), λ = 270 nm.

2.2.4 Synthesis of probe **L4**([Ding D, 2013](#_ENREF_1))

In a 50 mL two-neck round-bottom flask, Compound **4b** (100.00 mg，286.98 μmol) and compound c (122.32 mg，315.67 μmol) were dissolved in a mixture of t-BuOH/H_2_O solution (v/v = 2/1; 27 mL), and the reaction was shaken for a few minutes to obtain a clear solution. The “click” reaction was initiated by sequential addition of the solution of sodium ascorbate (0.1 M, 8.4 mL) and CuSO_4_ (0.1 M, 2.4 mL) in water. The reaction was continued with stir at 50 °C for another 1 h. The reaction mixture was evaporated in vacuo and was extracted with DCM. The organic layer was collected and concentrated. The crude product was purified by silica-gel chromatography, then added ethyl acetate saturated with hydrogen chloride to give probe **L4** as yellow-brown solid in 12% yield. M.p.: 62-65 °C.

^1^H-NMR (100 MHz, DMSO-*d_6_*): δ ppm: 10.44 (s, 1H), 8.00-7.94 (m, 3H), 7.33-7.30 (m, 2H), 7.16-7.08 (m, 9H), 7.05-7.02 (m, 2H), 6.98-6.94 (m, 8H), 5.48 (s, 2H), 3.65-3.63 (m, 1H), 3.54-3.51 (m, 2H), 3.12 (s, 3H), 3.09-3.07 (m, 4H), 2.71-2.66 (m, 2H), 2.08-2.04 (m, 2H), 1.96-1.91 (m, 4H).

^13^C-NMR (400 MHz, DMSO-*d_6_*): δ ppm: 199.86, 146.12, 143.79, 143.46, 140.42, 131.38, 131.04, 131.01, 130.59, 128.36, 128.32, 128.26, 127.64, 127.04, 122.93, 118.08, 60.22, 56.03, 52.78, 51.44, 26.19, 23.43, 22.72, 21.24, 14.56.

ESI-HRMS: ([M+H]^+^) calcd for C_45_H_45_N_5_O_3_S: 736.3316, found: 736.3393.

HPLC, tR = 5.628 min, mobile phase: methanol-water (97:3, v/v), λ = 270 nm.

**3. Fluorescent excitation and emission spectra of free probe L1-L4**


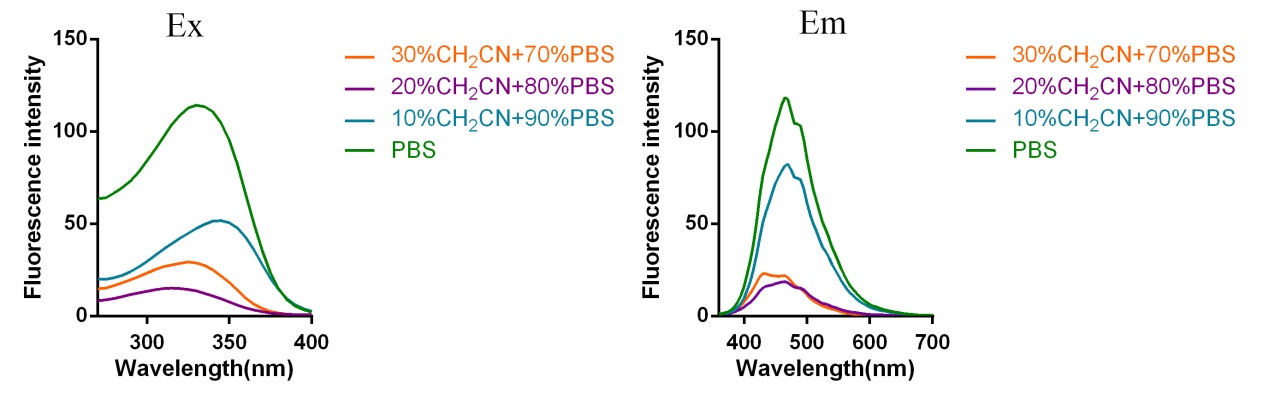


Figure S1. Fluorescent excitation (the emission wavelength was 465 nm) and emission spectra (the excitation wavelength was 330 nm) of probe **L3** in solution with different ratio of acetonitrile and PBS.


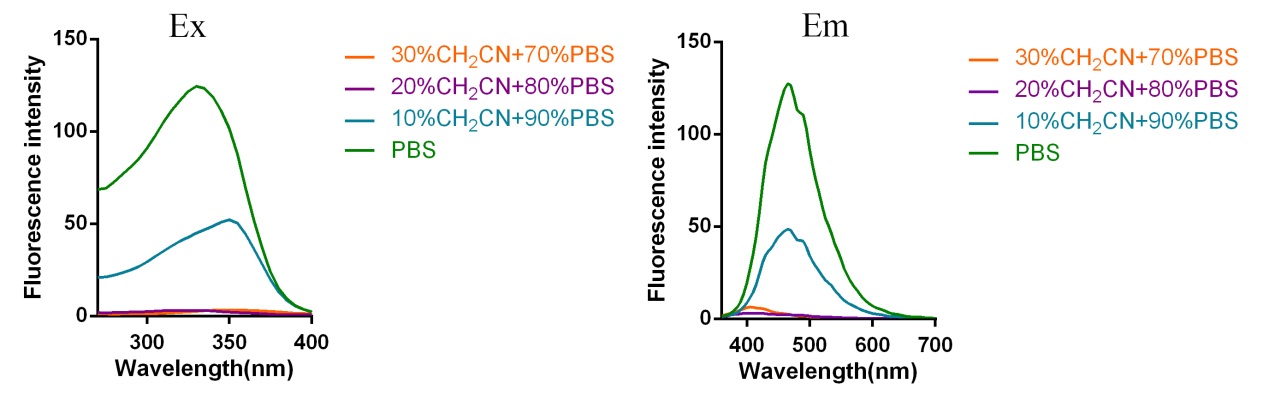


Figure S2. Fluorescent excitation (the emission wavelength was 465 nm) and emission spectra (the excitation wavelength was 330 nm) of probe **L**4 in solution with different ratio of acetonitrile and PBS.

**4. The quantum yields measurement**

The probes **L1-L4** (10 mM) was diluted with PBS (pH =7.4) into dilute solutions of different concentrations (1-5 μM). The standard (10 mm x 10 mm) quartz sample pool with white PTFE plugs was used in the determination, one for PBS (pH =7.4) and one for the solution of probe. Then, the quantum yields were determined by combined steady state/transient fluorescence spectrometer (FLS920).

**5. hERG potassium channel inhibition assay**


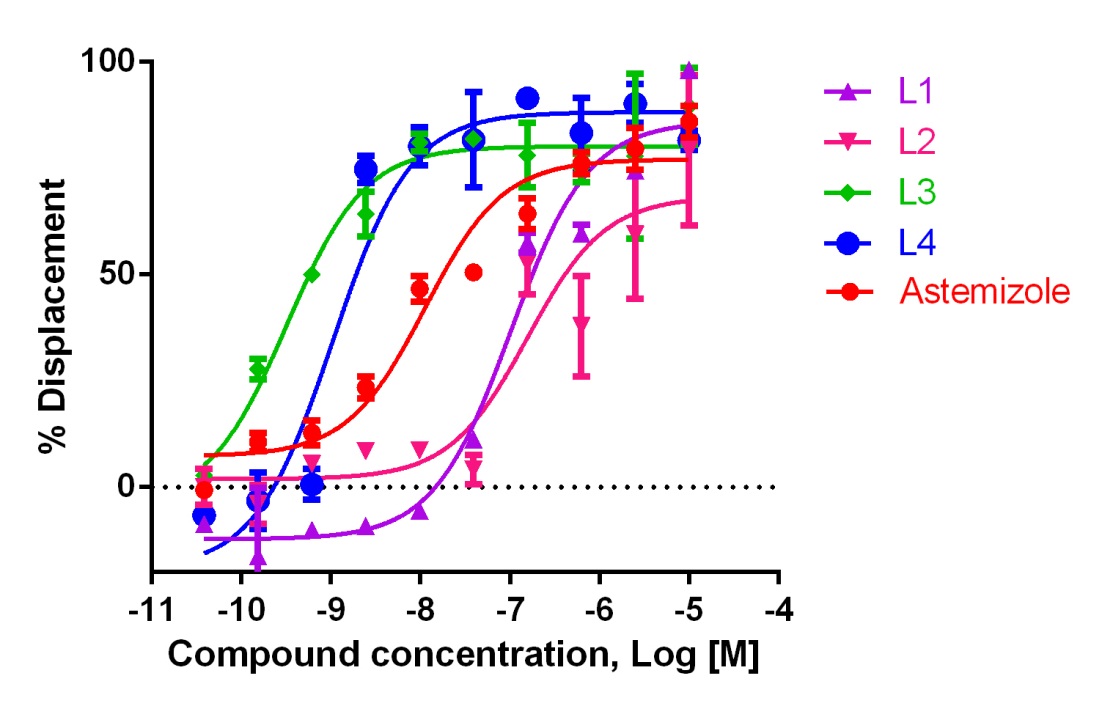


Figure S3. Competitive binding curve of probe **L1-L4** and astemizole to hERG potassium channel was determined by radio-ligand binding assay.

The inhibitory activity on hERG potassium channel was determined by a radio-ligand binding assay. Astermizole (Cat. No.#A2861-10MG, Sigma-Aldrich) was chosen as positive contral. The affinity with hERG potassium channel was assessed in the presence of 7.5 nM ^3^H-dofetilide (Perkin Elmer# NET1144100UC). The probe’s binding abilities with the hERG potassium channel were displayed with displacement curves and compared to the positive controls. In brief, probe **L1-L4** and astemizole were dissolved in DMSO as stock solution (10 mM), which was further diluted with binding assay buffers when applied to the binding assays. Cell membranes were prepared in HDB. First, each well of Uni-filter 96 GF/B microplate was incubated with 80 μL hERG membrane (10 μg protein/well), 10 μL of ^3^H-dofetilide (7.5 nM final) and 10 μl of compounds (10 points, 4 fold dilution from 10 μM) in binding assay buffer at 37 ^0^C for 1.5 h. Then stop the binding reaction by rapid filtration through GF/B plates using cell harvester. The wells was washed with cold wash buffer for three times and dried at 37 ^0^C for 30 minutes. 50 μL scintillation cocktail was added to each well. Radioactivity was determine by MicroBeta Trilux (Perkin Elmer 1450). Data were recorded by Topcount NXT and stored on the GenScript computer network for off-line analysis. Data acquisition was performed by Microsoft Excel (version 2010) program; IC_50_ values were obtained by GraphPad Prism 6 using the Cheng-Prusoff equation. The binding data was converted to %displacement according to the below equation: %displacement=100 × (1-(sample CPM/Total binding CPM)) (in which total binding CPM values were obtained by testing binding of ^3^H-dofetilide to the targets without competitors).

**6. Cytotoxicity**

The cytotoxicity effects of probes were determined by Cell Counting Kit-8 assays, using hERG transfected HEK293 cells (hERG transfected HEK293 cells were purchased from Shanghai Genechem Co., Ltd). An amount of 5×10^3^ cells per well were seeded in 96-well plates in 100 μL culture medium and cultured in 5% CO_2_ atmosphere at 37 °C for 24 h. Then, the cells were treated with 100 μL of different concentrations solutions of each probe (**L1**, **L2**, **L3**, or **L4**) for 24 h, respectively. Subsequently, 20 µL of CCK-8 solution was added to each well, and then the plates were incubated for 2 h at 37 °C. After 2 h, the absorbance values of the wells were recorded using a microplate reader at 450 nm. Wells containing no probes were chosen as blanks. Then, the half maximal inhibitory concentration (IC_50_) of each probe was calculated by GraphPad Prism 6.

**7. Cell culture and fluorescence microscopy imaging**

hERG transfected HEK293 were grown in DMEM medium supplemented with 10% (v/v) fetal bovine serum in an atmosphere of 5% CO_2_, 95% air at 37 °C. Cells were plated on confocal dish and allowed to adhere for 12 h~24 h. After the medium was removed, the cells were carefully washed with DMEM medium without fetal bovine serum, and then incubated at r.t. in the presence of the probe **L1-L4** (prepared in DMEM medium without fetal bovine serum) or co-incubated with L1-L4 and astemizole (a potent hERG channel blocker) for 10 min. Fluorescence imaging was performed using Zeiss Axio Observer A1 fluorescence microscope.


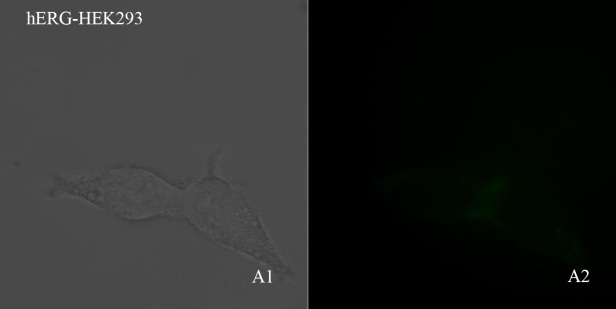

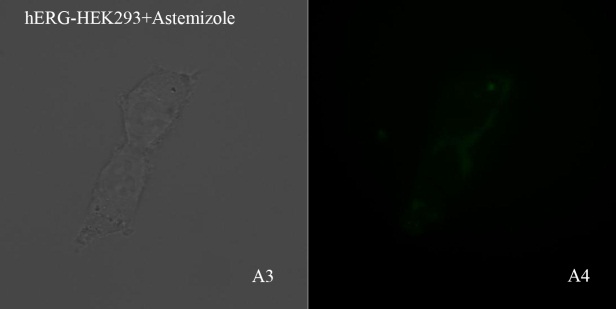


Figure S4. Fluorescence microscopic imaging of hERG transfected HEK293 cells in absence or presence of 10 µM Astemizole (A1,A3: bright field, A2, A4: GFP channel). Performed in Zeiss Axio Observer A1; Objective lens: 63×

In addition, further investigation has been done to detect whether the cells used in the current study have an autofluorescence, or astemizole can affect the autofluorescence of cells. In this assay, we incubated the cells with astemizole (10 µM, prepared in medium without fetal bovine serum) at 37 °C in an atmosphere of 5% CO_2_, 95% air for 10min. Then, fluorescence imaging of hERG-HEK293 cells was obtained in presence or absence of astemizole. The results displayed that the autofluorescence of cells is so weak in absence and presence of astemizole (Figure S5), which would not influence the imaging of cells using the obtained probes L1-L4.

**8. ^1^H-NMR, ^13^C-NMR, ESI-HRMS, HPLC.**


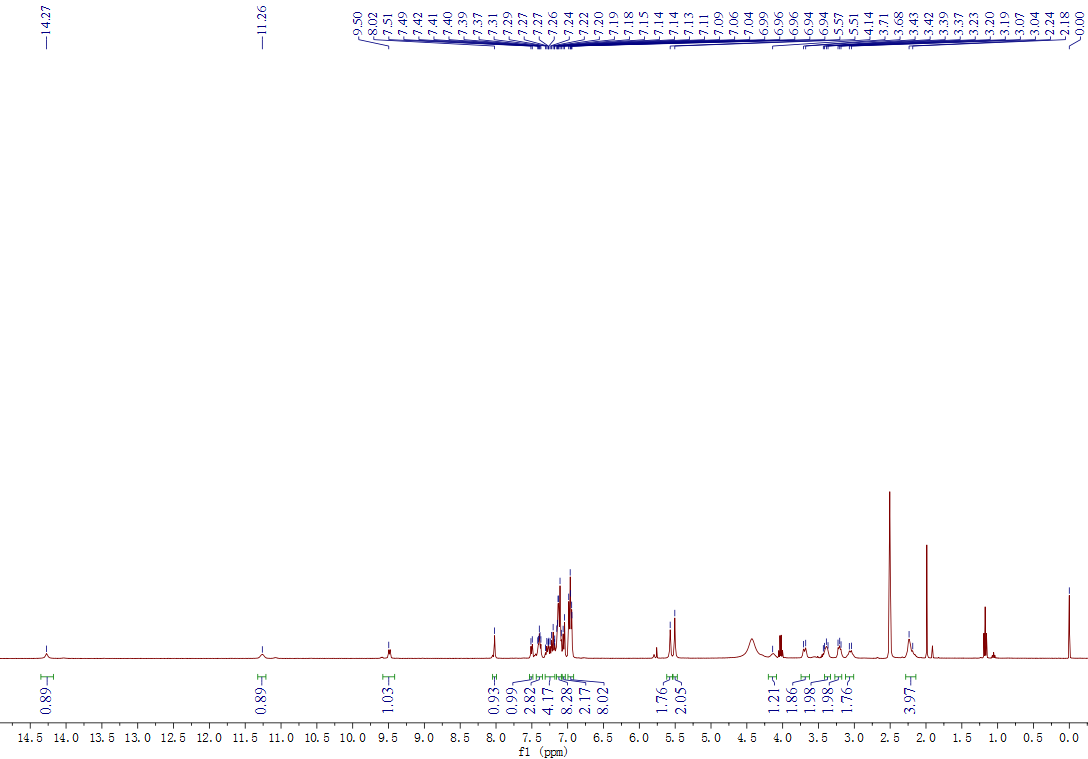


Figure S10. ^1^H-NMR spectrum of compound L1.


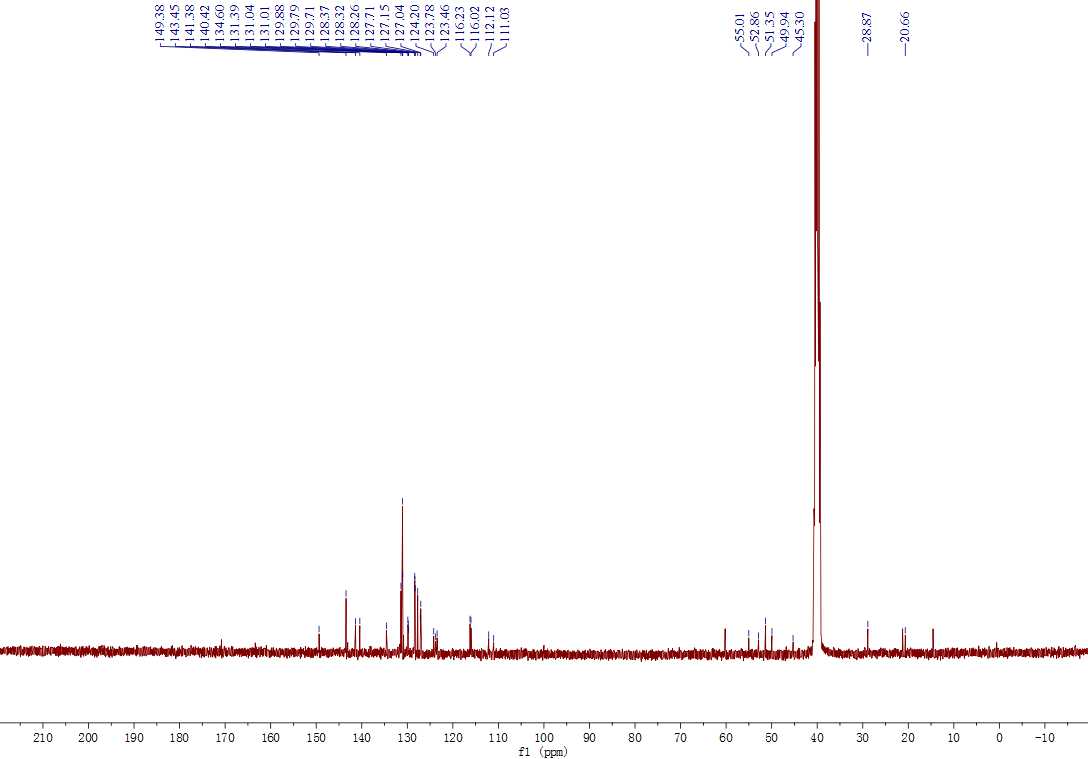


Figure S11. ^13^C-NMR spectrum of compound L1.

Figure S12. ESI- HRMS spectrum of compound L1.


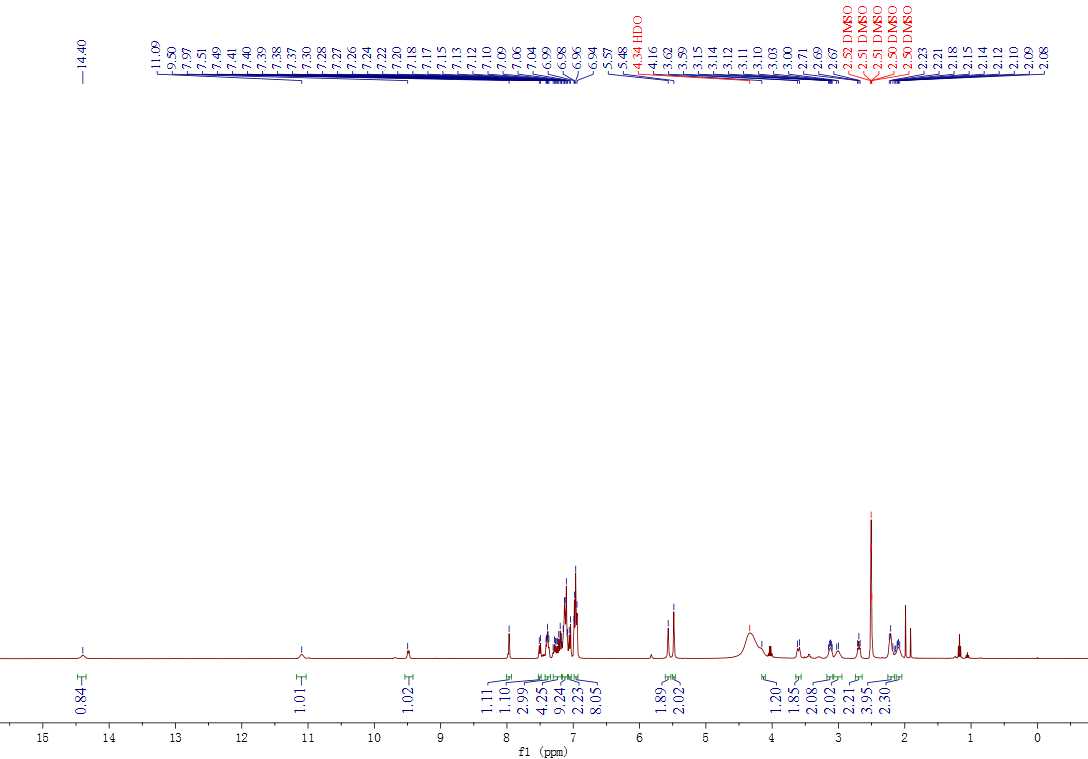


Figure S10. ^1^H-NMR spectrum of compound L2.


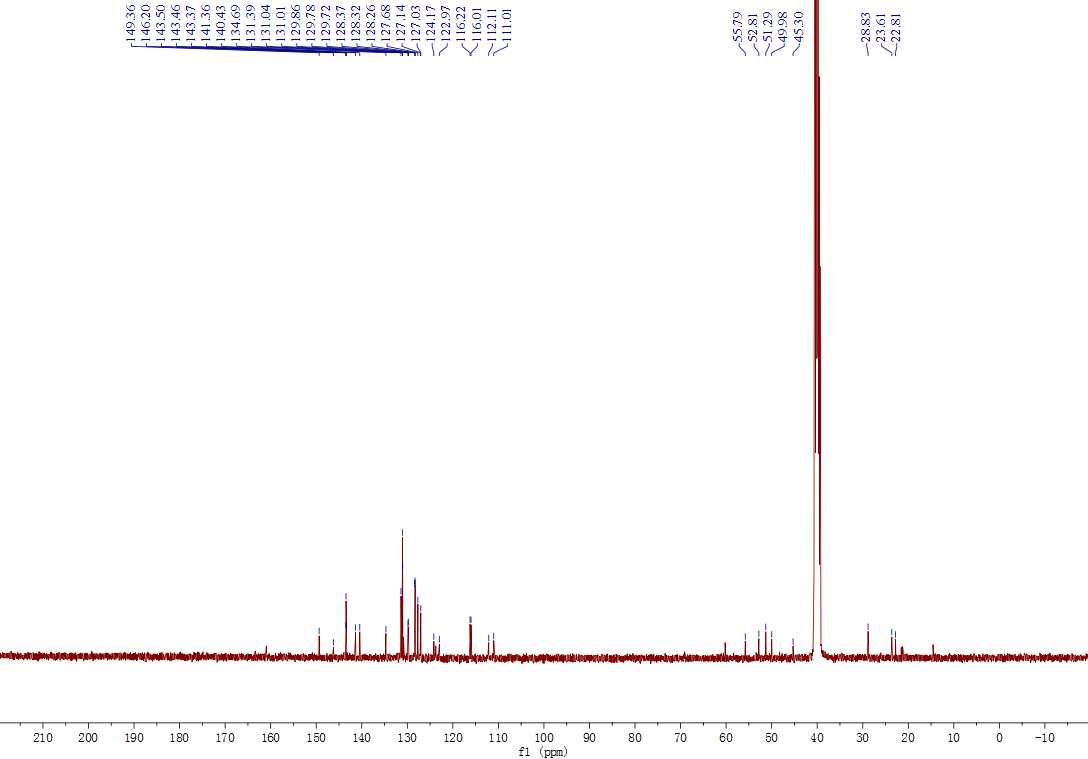


Figure S11. ^13^C-NMR spectrum of compound L2.

Figure S12. ESI- HRMS spectrum of compound L2.


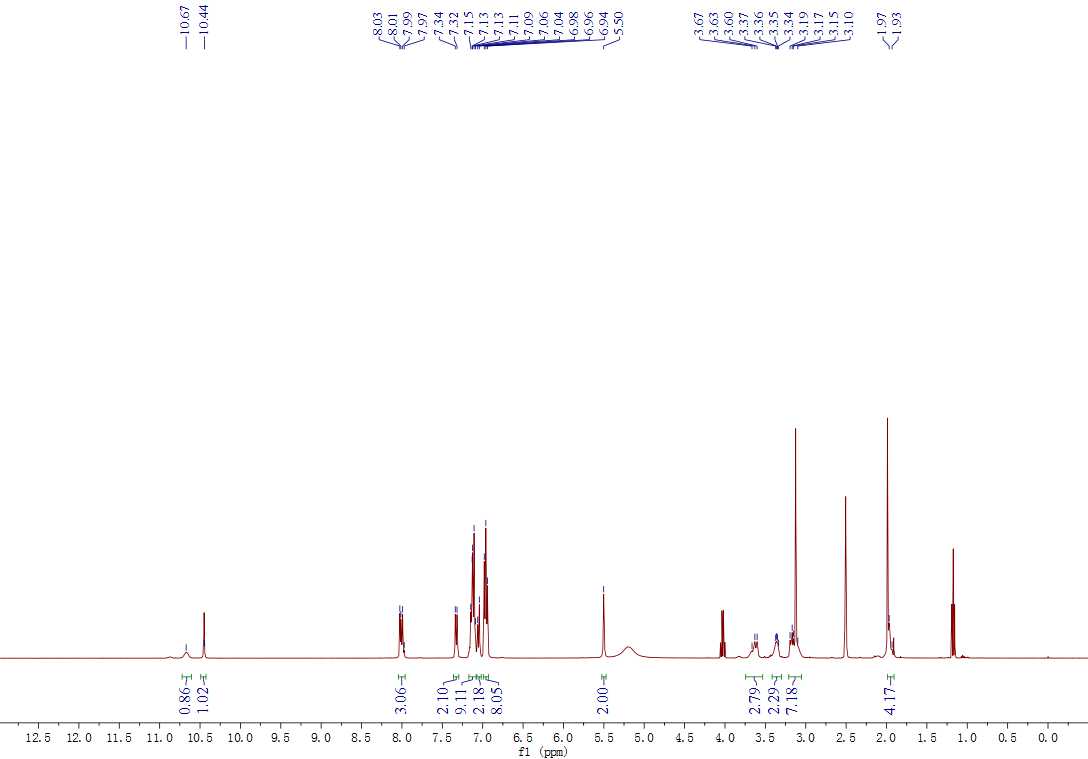


Figure S10. ^1^H-NMR spectrum of compound L3.


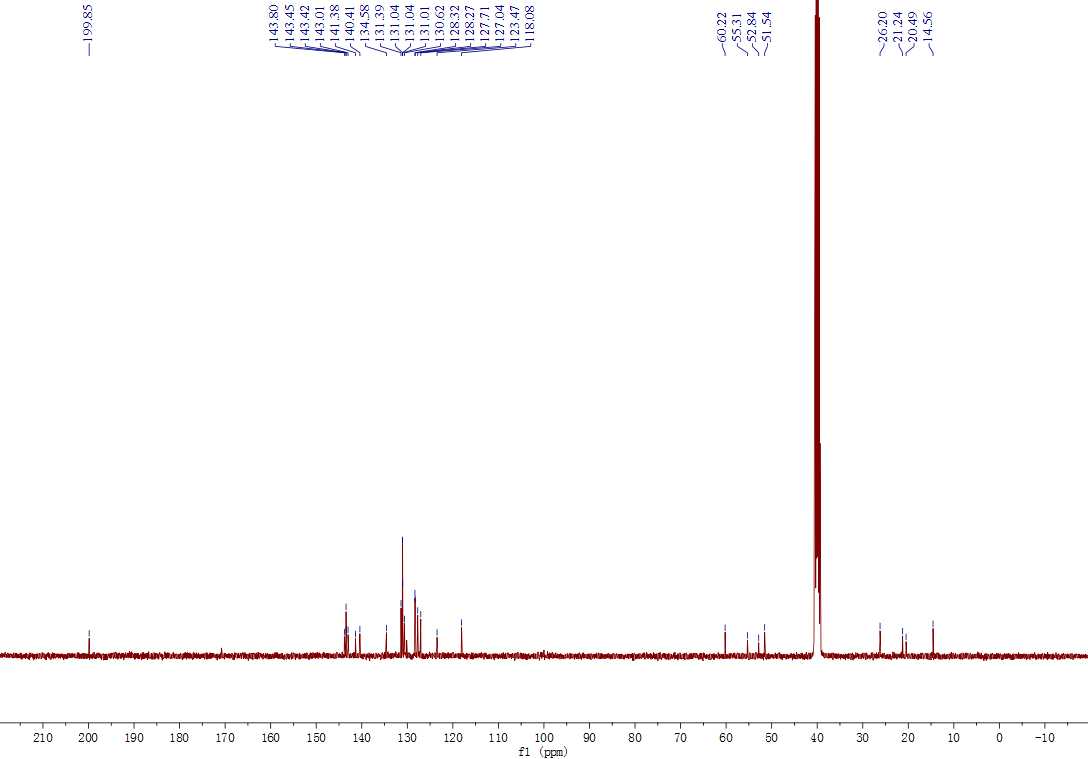


Figure S11. ^13^C-NMR spectrum of compound L3.

Figure S12. ESI- HRMS spectrum of compound L3.


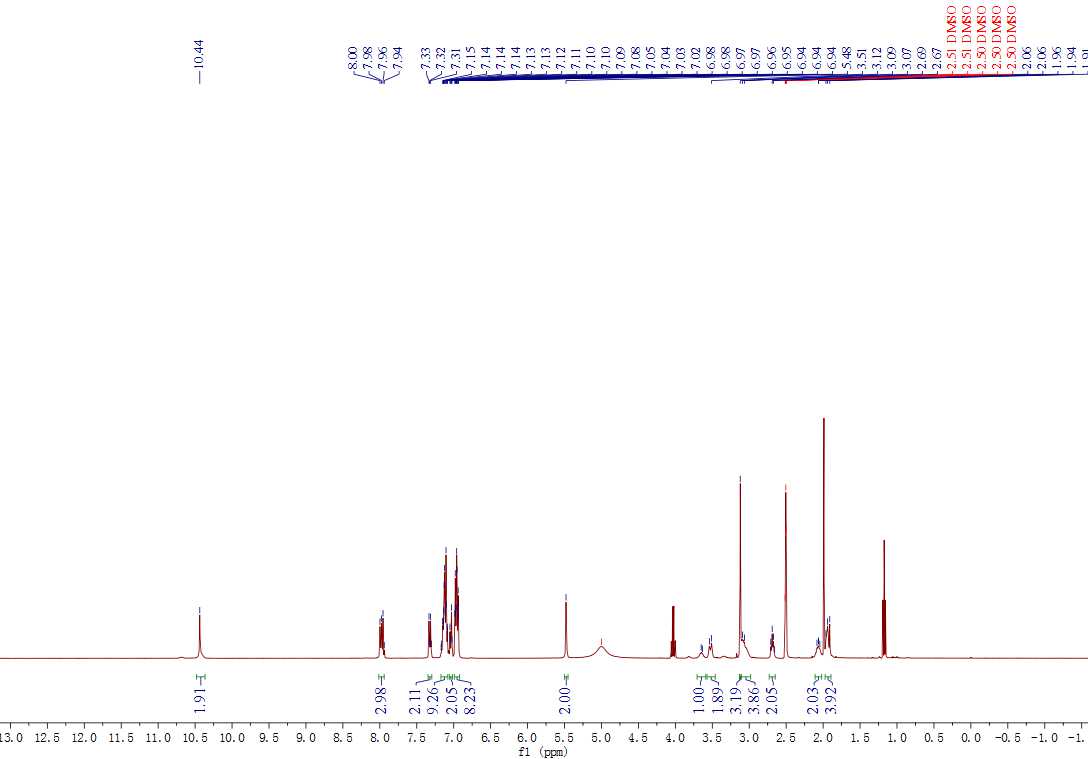


Figure S10. ^1^H-NMR spectrum of compound L4.


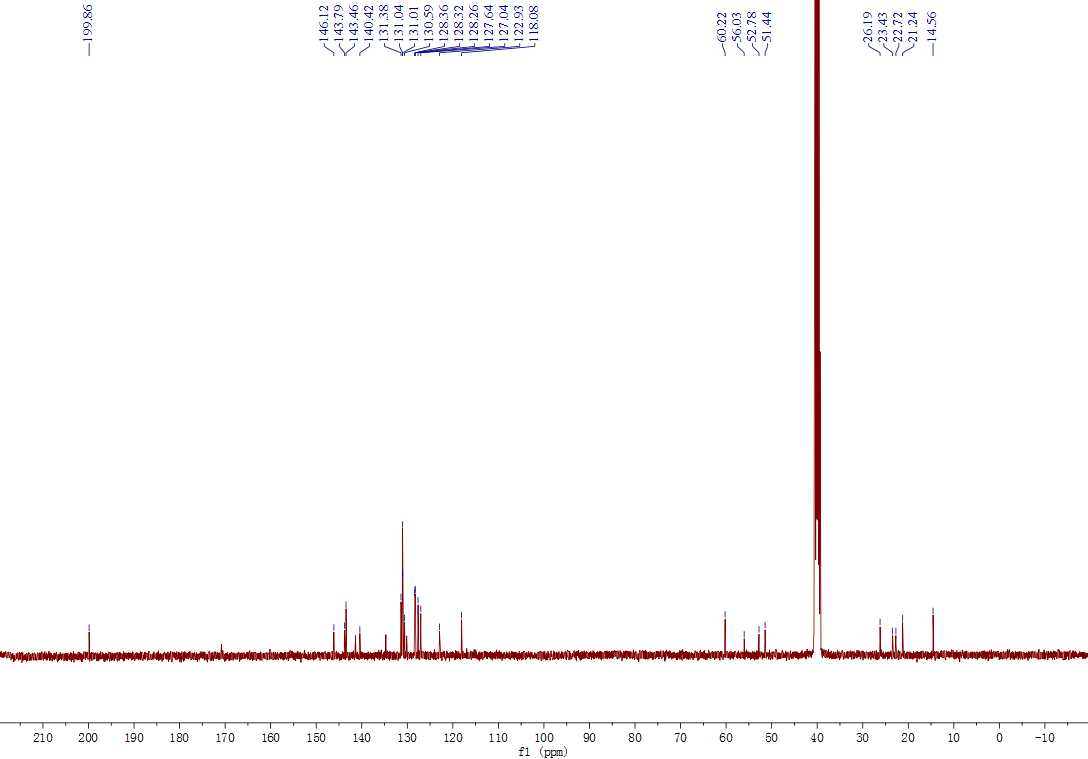


Figure S11. ^13^C-NMR spectrum of compound L4.

Figure S12. ESI- HRMS spectrum of compound L4.

HPLC assessment of compound purity.

All tested compounds (1a, 1b, 1c) with a purity of >95% were used for subsequent biological assays. We provided the spectra of HPLC assays as below.

L1, 96.50%


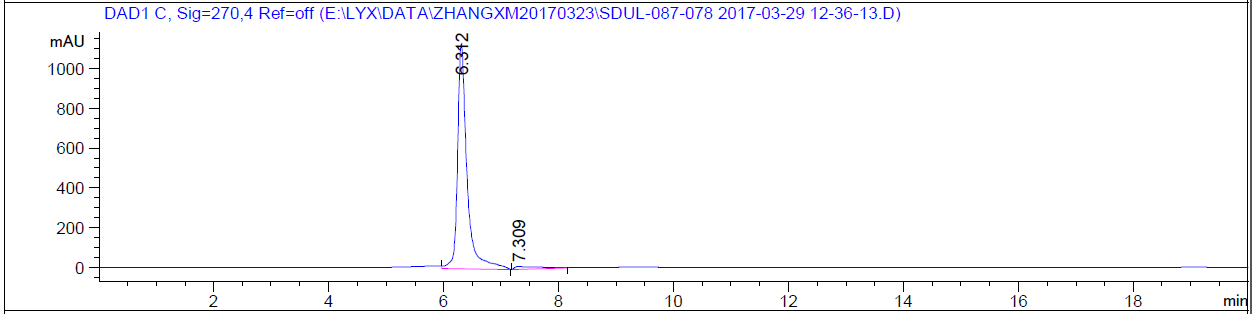


L2, 99.73%


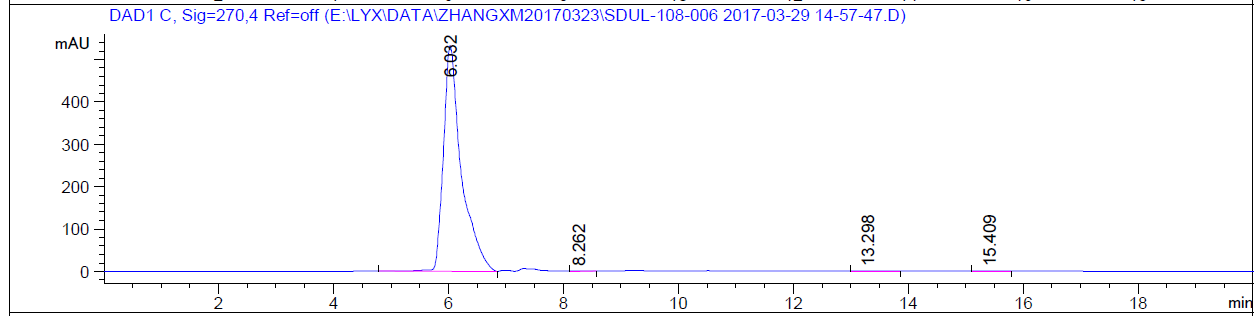


L3, 98.06%


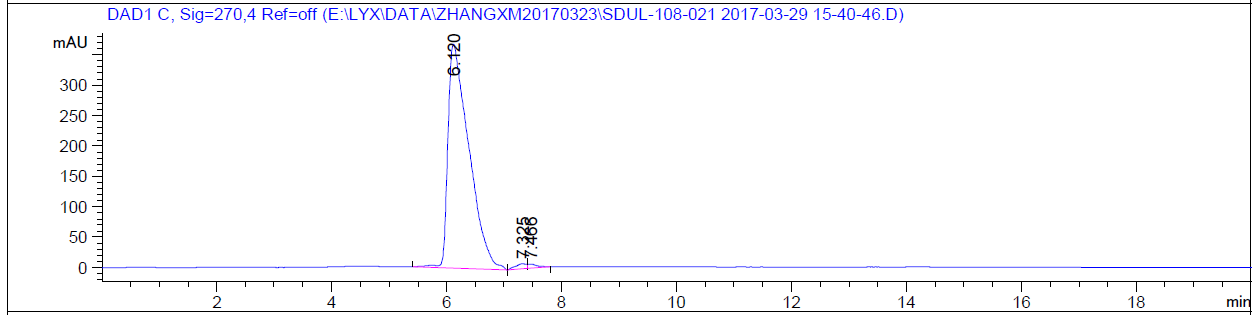


L4, 96.25%


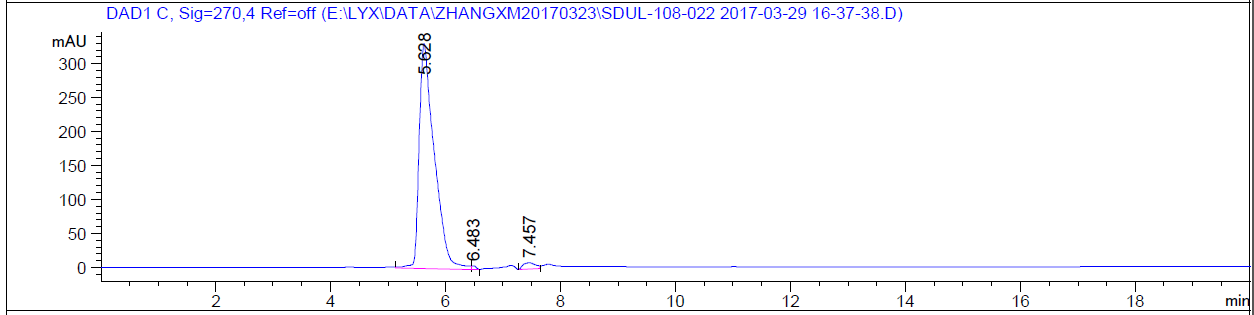


**9. Reference**

Ding D, L.K., Liu B, Et Al. (2013). Bioprobes based on AIE fluorogens. *Accounts of Chemical Research* 46**,** 2441-2453.

Liu, Z., Jiang, T., Wang, B., Ke, B., Zhou, Y., Du, L., and Li, M. (2016). Environment-Sensitive Fluorescent Probe for the Human Ether-a-go-go-Related Gene Potassium Channel. *Anal. Chem* 88**,** 1511-1515.

**
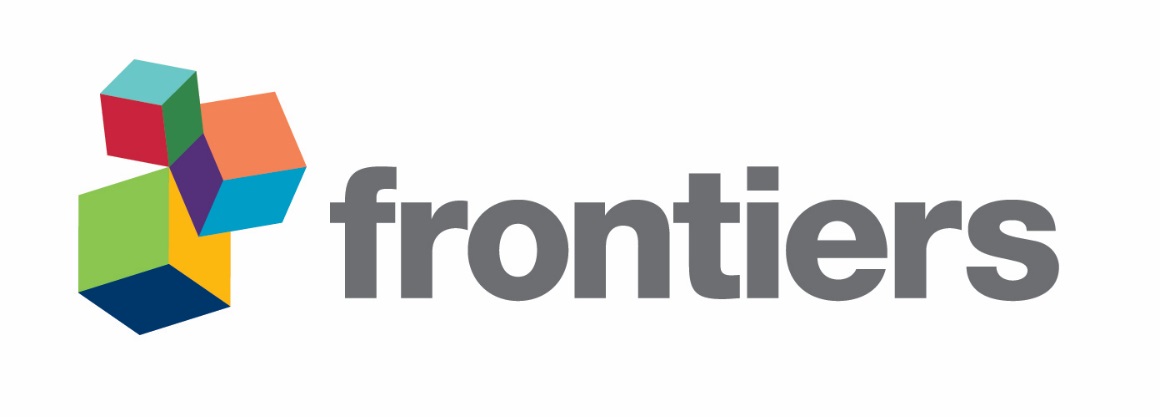
**
